# Lead Toxicity From a Swallowed Fishing Sinker: A Case Report

**DOI:** 10.1097/PG9.0000000000000084

**Published:** 2021-06-25

**Authors:** Shivani R. Gupta, Ethan Mezoff, Molly Dienhart

**Affiliations:** From the Department of Gastroenterology, Hepatology and Nutrition, Nationwide Children’s Hospital, Columbus, OH.

**Keywords:** lead, poisoning, ingestion, foreign body, endoscopy

## Abstract

Lead toxicity is relatively common despite increased public awareness, with lifelong neurologic sequelae. Common sources of exposure include lead paint, contaminated water, curtain weights, and bullets. However, few reports consider management of lead fishing equipment ingestions, such as weights or sinkers. We present a 5-year-old male who swallowed a lead fishing weight and had a high serum lead level despite urgent removal. When managing ingestion of a lead foreign body, if accessible by upper endoscopy, we recommend urgent removal with subsequent monitoring of serum lead levels.

**What Is Known**

- Lead toxicity can cause long-term neurologic sequelae.
- Lead ingestion can cause serious side effects including gastritis and lead toxicity.
- In addition to observation and serum lead level monitoring, several interventions are available to treat a high lead level.

**What Is New**

- Following ingestion, we suggest urgent endoscopic removal over whole bowel irrigation if the lead object is reachable.
- We suggest initiating a proton-pump inhibitor while the object is within the gastrointestinal tract.
- We suggest consultation with toxicology for timing of repeat lead levels and to assess whether chelation therapy would be beneficial.

## INTRODUCTION

In children, lead toxicity causes intellectual disability, hearing impairment, encephalopathy, and peripheral nerve impairment (1). In the early 1990s, a serum lead concentration over 10 µg/dL in children was common (normal < 5 µg/dL) (2), secondary to common household items that contained lead. Over the last several decades, conscious public efforts have helped to reduce obvious environmental sources of lead, including paint, dust, and soil (2). Despite these efforts, many household and sporting items still contain lead. Over 2000 cases of ingestions with small lead items were reported to poison control in 2016 (3,4). These items were not specified; however, 38 of those cases were recorded as fishing equipment and the majority occurred in children under 6 years of age (3,4). In 2018, there were 47 cases of ingestions with fishing baits and an additional 13 with other miscellaneous fishing products (5). We present a case highlighting that exposure to lead-containing hobby equipment, such as lead bullets and fishing sinkers, should also be considered. Most fishing sinkers are made of solid lead as it is inexpensive and easy to mold. States including California, Maine, Massachusetts, New Hampshire, Vermont, and New York have banned the use of lead fishing weights out of concern for their potential harm to wildlife. Few reports discuss lead toxicity from a fishing weights or sinkers or guide their endoscopic removal. We report a pediatric patient who ingested a lead-containing fishing weight and had a resultant elevated serum lead level. Informed consent was obtained from the family for this report.

## CASE REPORT

**FIGURE 1.**
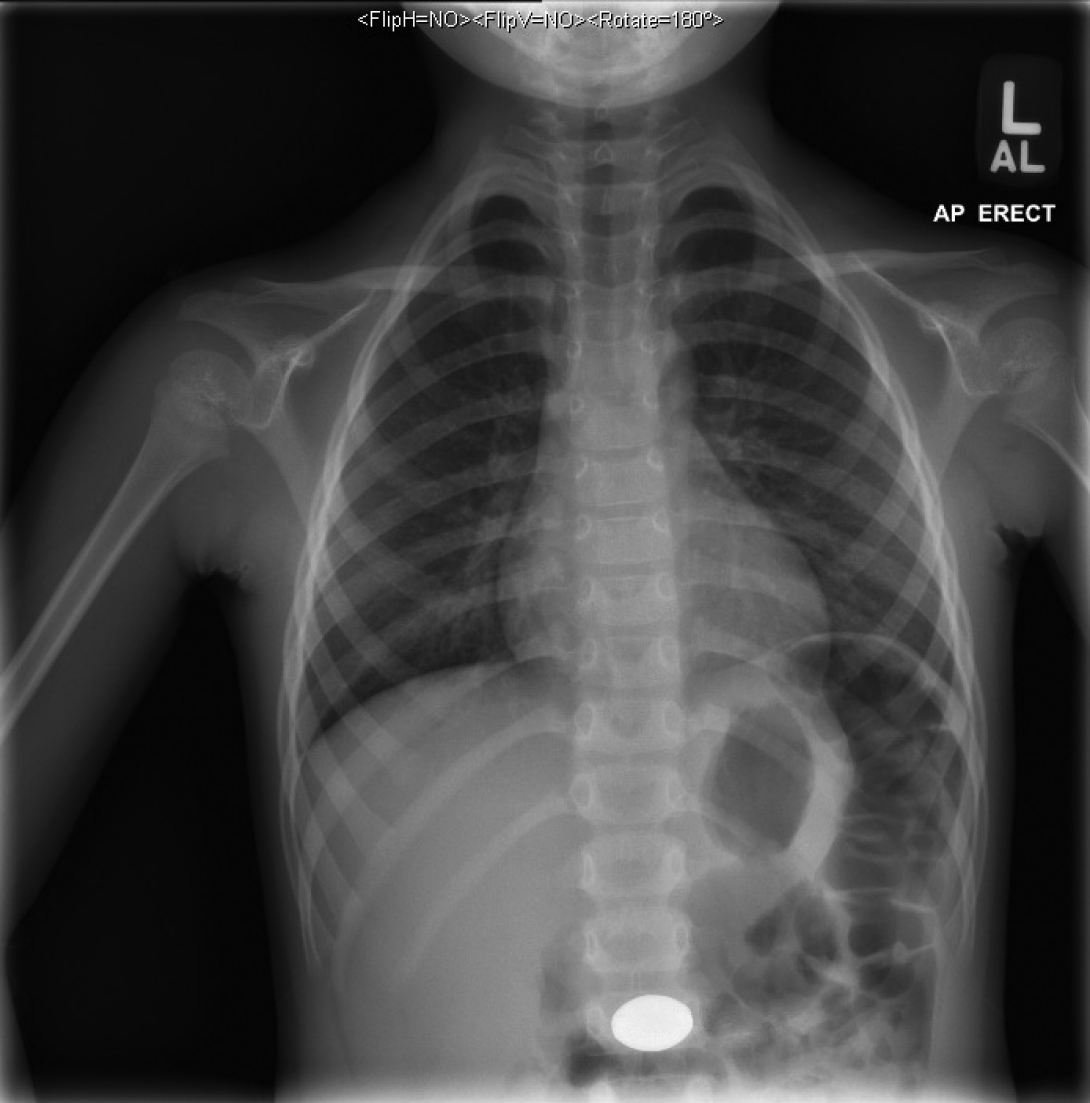
Chest x-ray obtained upon presentation to the local hospital. A radiopaque foreign body is seen in the mid-abdomen. AL = anterolateral; AP = anteroposterior; L = left.

A 5-year-old healthy male presented to the emergency department (ED) with his mother after the ingestion of a fishing weight. Around 4 pm on the day of presentation, he was playing with 2 weights. After some time, mom noticed that he only had one left. She immediately took him to their local hospital, where he had an upright anteroposterior chest x-ray that showed a 2.3 × 1.5 cm radiopaque foreign body in his mid-abdomen (Fig. 1). He was transferred to the pediatric hospital for further management. Throughout this time, the patient had no nausea or vomiting but complained of diffuse abdominal pain. Vital signs, respiratory status, and abdominal exam were normal. Poison control was notified and recommended that the patient be made nil per os with initiation of pantoprazole and a serum lead level. The ED physicians and poison control considered bowel irrigation versus immediate removal of the weight, and pediatric gastroenterology was consulted.

**FIGURE 2.**
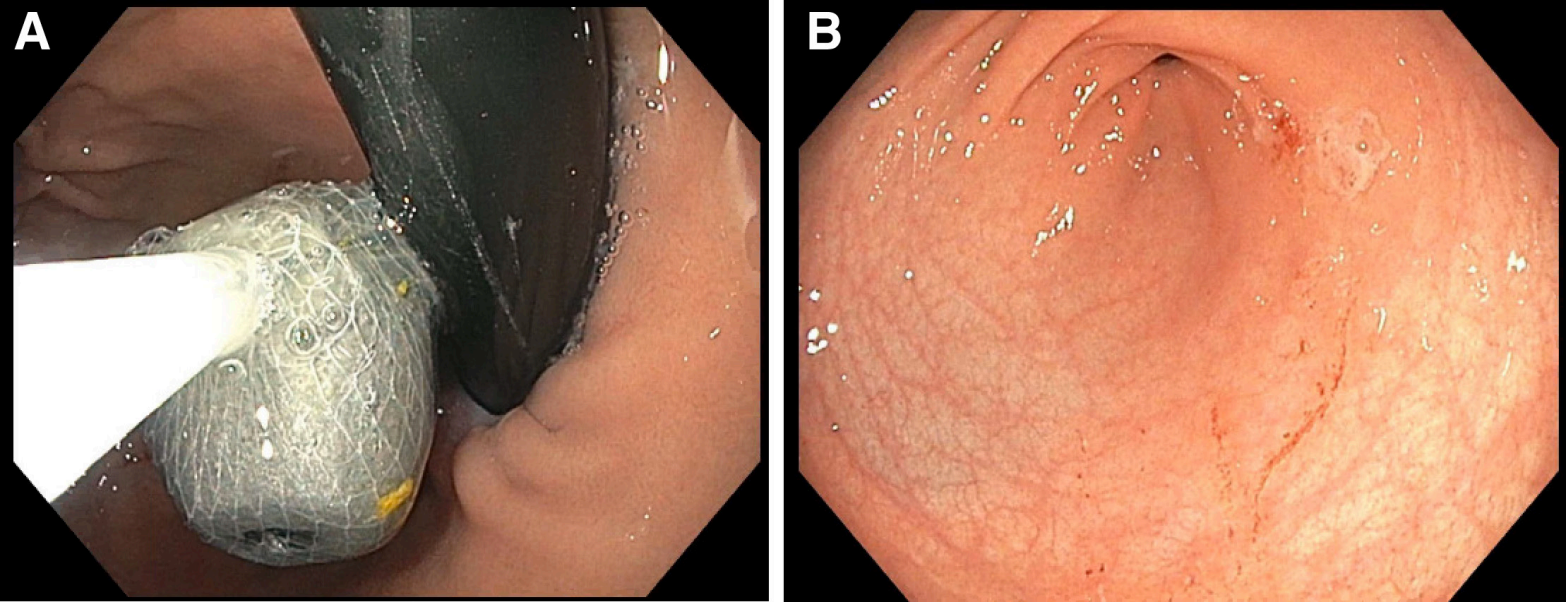
Endoscopic images of foreign body and pylorus. A) The lead weight was found in the gastric fundus and removed using a retrieval net. B) Mild erythema and irritation found in the gastric antrum after removal of the fishing weight. The patient did not have any other visual complications from the ingestion by EGD. EGD = esophagogastroduodenoscopy.

Repeat supine abdominal x-ray indicated the weight’s likely intragastric location. Review of available literature described poor antegrade propagation with bowel irrigation (3). Therefore, he was taken to the operating room approximately 6 hours after the ingestion. The weight was successfully removed with a retrieval net (Fig. 2A) without complication. Mild erythema and irritation but no ulcerations were noted in the gastric antrum (Fig. 2B). He was admitted overnight for observation to ensure recovery from anesthesia and was discharged following tolerance of oral intake. His lead level was pending at the time of discharge.

The lead level collected approximately 5 hours after the ingestion, reported the following afternoon, was 68 µg/dL. Without other known exposures, toxicology recommended observation with a repeat level in 1 week. The expectation was that the level would decrease spontaneously with removal of the source. Three days later, the level was 39 µg/dL. Three months after initial ingestion, the level was 14 µg/dL without any intervention. The trend was felt to be appropriate, and the toxicology consultant recommended a repeat level in 3 months. Unfortunately, the family was nonadherent to this recommendation, and no additional levels have been obtained.

## DISCUSSION

Our case documents an acute rise in lead level due to ingestion of a lead fishing weight that resolved with time. While our patient did not display any signs of acute lead toxicity, chronic exposure and chronically elevated lead levels can cause lasting cognitive impairment. Therefore, minimizing lead exposure to young children is critical. The most effective way to prevent lead exposure is to eliminate the environmental elements that pose risk, which may contain household items.

Mowad et al (6) reported an 8-year-old who ingested more than 20 sinkers. The sinkers were beyond the stomach, prompting whole bowel irrigation until only 3 sinkers remained and were beyond the reach of a standard endoscope (6). This patient’s initial lead level was 53 µg/dL and normalized to 4 µg/dL about a month later with oral Succimer (6). Another case report described improvements in lead levels with oral Succimer after ingestion of a lead weight (7). Lead increases oxidative stress on the stomach, leading to gastric ulcers; therefore, proton-pump inhibitors are recommended (8). In our patient, the lead weight was in his stomach for only 5–6 hours prior to its removal, but his lead level was over 12 times the upper limit of normal. While other reports do not specifically address timing between ingestion and significant elevation of serum lead level, the relatively short interval in our patient was concerning for other potential exposures to lead, such as living in an older home. Previous lead levels were not available for comparison, however, per father’s report, he had no known history of elevated lead levels prior to the ingestion. The family had recently moved from New Mexico, where their house was built in the early 2000s. Further, their Ohio residence was built in 2010 suggesting low likelihood of lead exposure due to paint or building materials. His complete blood count at the time of presentation was unremarkable and inconsistent with chronic exposure.

Laboratory reporting of lead levels can be prolonged (for our patient, 17 h) and may not be useful for guiding more acute management decisions. Our patient was ultimately not treated because he was asymptomatic, and lead levels were trending downward after removal of the lead weight.

Unfortunately, despite recommendations from poison control, we were not able to follow the lead level down to a normal range after the ingestion of the weight. He subsequently presented to the ED on 2 occasions for other complaints. In his visit in October 2020, he presented for lymphadenopathy and had a complete blood count that was normal, but a repeat lead level was not performed.

The Ohio Department of Health has recorded the prevalence of confirmed elevated serum lead level ≥10 µg/dL in Ohio children (< 6 y old) from 1999 to present (9). Specifically, since 2013, the prevalence of a serum lead level ≥10 µg/dL has been <1%, and since 2015, the prevalence of serum lead levels ≥5 µg/dL has been about 2%. It is believed that >60% of housing in Ohio was built prior to 1980 and likely contained some lead-based paint; however, <3% of Ohio children less than 6 years of age were found to have elevated lead levels (9).

The North American Society for Pediatric Gastroenterology, Hepatology, and Nutrition clinical report offers management recommendations for removal of ingested foreign bodies in children, but lead-containing objects are not addressed (10). Lead objects do warrant special consideration as they can cause high serum lead levels even if they remain in the body for short periods of time. It should be noted that detectable lead levels below the abnormal range are not necessarily harmless and have been shown to cause intellectual impairment. Whole bowel irrigation has shown variable effectiveness and medical intervention with chelation often fails to prevent harmful effects of lead poisoning (11,12). Therefore, if within an area accessible by upper endoscopy, the lead weight should be removed urgently, and lead levels should be monitored.

## ACKNOWLEDGMENTS

S.R.G. is a pediatric gastroenterology (GI) fellow at Nationwide Children’s Hospital, and she drafted and edited the article. E.M. is a faculty member of the GI department at Nationwide Children’s Hospital. He edited the article, approved the final article, and was involved in idea development. M.D. is a faculty member of the GI department at Nationwide Children’s Hospital. She edited the article, approved the final article, and was involved in idea development.
